# Minimally Invasive Correction of Prolapsed, Gangrenous Distal Limb of Loop Ileostomy to End-Loop Stoma

**DOI:** 10.1155/2020/8873388

**Published:** 2020-11-06

**Authors:** John Shelton, Sittampalam Rajendra

**Affiliations:** University Surgical Unit, Teaching Hospital, Jaffna, Sri Lanka

## Abstract

**Introduction:**

Prolapse can be a complication of loop stomas. A prolapsed stoma which cannot be reduced or complicated with strangulation needs surgical correction. This case report describes a minimal access correction of a prolapsed gangrenous distal limb of ileostomy. *Presentation of Case*. A 67-year-old male patient was diagnosed with a lower rectal carcinoma, staged T3N1M0. Following neoadjuvant chemoradiation, he underwent a laparoscopic anterior resection with a defunctioning loop ileostomy. One month later, he presented with prolapse of the distal limb of the ileostomy. The limb was gangrenous and the gangrenous part was removed by using a linear GI stapler, and the loop ileostomy was converted to end-loop ileostomy. *Discussion*. It is a simple and technically feasible method for treating a prolapsed loop of the stoma. It is less invasive and has minimal postoperative complications. This technique reduces the duration of the hospital stay of the patient.

**Conclusion:**

Stapled assisted correction of prolapsed stoma avoids unnecessary laparotomy and aids in expedite recovery after surgery. It is beneficial for a surgeon to be familiar with the minimal access correction for stoma prolapse.

## 1. Introduction

Diversion of faeces from the anastomotic site is achieved by creating a stoma proximally. Prolapse can be a complication of loop stomas. Prolapse occurs more frequently with loop stomas than end stomas and most frequently involves the distal limb. Patient-related risk factors for stoma prolapse include advanced age, obesity, increased intra-abdominal pressure, COPD, bowel redundancy, and weak fascia. Technical factors leading to prolapsed stoma are improper stoma site outside the rectus muscle, oversized aperture, and redundancy of the distal bowel at the stoma site [1–6].

Techniques that can limit stoma prolapse include extraperitoneal tunneling, mesentery-abdominal wall fixation, and limiting the size of the aperture. Symptoms associated with stoma prolapse include pain, skin irritation, and difficulty with maintaining an appliance and can rarely lead to obstruction, incarceration, or strangulation. Acute stoma prolapse can often be reduced at the bedside with the aid of sugar and ice to reduce bowel wall edema, allowing for an elective repair if prolapse was to recur. Surgical options for stoma prolapse include reversal of a temporary stoma (when possible and feasible), resection, revision, or relocation [7].

A prolapsing loop stoma can be remedied by converting it into an end-loop configuration. Loop stoma conversion to an end loop stoma is performed by incising the mucocutaneous junction and transecting the bowel used to create the loop stoma into a distal and proximal segment. The prolapsed bowel segment, which tends to be the distal (efferent) limb, is closed [8–11]. Local dissection with linear cutting stapler can provide a unique and effective technique in addressing this issue when the stoma cannot be reversed. The first of this case was reported from Italy in 2003; the patient did not develop any postoperative recurrence or complications [12].

In this case report, we have proposed a minimally invasive correction of the prolapsed stoma to end-loop stoma by using a single liner cutting stapler, which is cost-effective and less morbidity to patients.

## 2. Presentation of Case

### 2.1. Patient Presentation

A 67-year-old Sri Lankan male patient, presented with altered bowel habit and bleeding per rectum for 18 months. A digital rectal examination revealed a rectal tumour. On colonoscopy, he was found to have a rectal mass of about 10 cm from the anal verge obstructing the lumen. The histopathology report of the biopsy from the lesion confirmed a moderately differentiated adenocarcinoma. A contrast-enhanced CT scan of the abdomen and pelvis staged the tumor as T3N1Mx.

The patient underwent neoadjuvant chemoradiotherapy and was found on restaging to have T2N0Mx tumor. He underwent standard laparoscopic low anterior resection with loop ileostomy.

**Figure 1:**
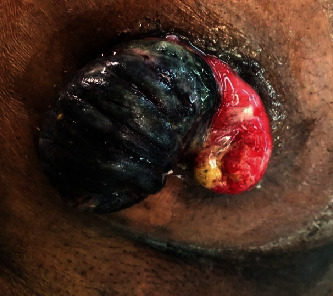
Photograph of distal loop prolapse of defunctioning loop ileostomy.

After 40 days of primary surgery, he presented with a prolapsed incarcerated distal loop of ileostomy. On examination, the prolapsed distal limb of loop ileostomy was gangrenous (Figure 1). The local revision of the prolapsed segment was planned, and informed written consent was obtained for local revision with possible conversion to laparotomy.

### 2.2. Intervention

**Figure 2:**
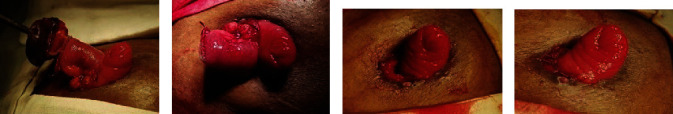
(a), (b) Prolapse distal limb stapled. (c) Distal limb reduced into the abdomen. (d) Corrected stoma prolapse.

The surgery was performed under general anesthesia and in the supine position. After the administration of preoperative antibiotics, the gangrenous prolapsed segment was examined and confirmed to be the distal limb of the ileostomy. As the prolapsed segment was gangrenous, no reduction under general anesthesia was attempted. The prolapsed distal loop was lifted up by using two Babcock forceps. A circumferential incision was made along the mucocutaneous junction of the gangrenous bowel loop using diathermy. Dissection was carried out to liberate the prolapsed distal loop from the anterior rectus sheath and peritoneal adhesions. The bowel used to create the loop stoma was separated into a distal and proximal segment by transecting the wall. Some more distal loop was delivered out, and a single fire of 80 mm (COVIDIEN 80 mm-4.8 mm—UK) linear stapler device was used to close the distal limb at about 1–2 cm distal to the gangrenous part (Figures 2(a) and 2(b)). The distal loop was kept inside the peritoneal cavity. The dissected part of the proximal bowel loop was anchored to the skin using a 2/0 polyglactin suture (Figures 2(c) and 2(d)).

The final appearance of ileostomy site after complete removal of the prolapsed segment and reconstruction is that of a loop ileostomy being converted to end-loop ileostomy [13]. There was minimal blood loss, and the surgery took only 25 minutes. The proximal end of the loop ileostomy was patent and functioning. After fixing the stoma appliances, the patient was recovered from anesthesia and was transferred to the ward. He had an uneventful postoperative recovery. In the postoperative period, no opioid analgesics were used as the patient had minimal pain, and oral intake was resumed on the day of surgery. Stoma function was not affected, and he was discharged home on postoperative day one.

In the weekly postoperative follow-up, he had no evidence for recurrent prolapse, obstruction, or other complications until the ileostomy reversal. After 18 weeks of primary surgery, he underwent for routine ileostomy reversal without any difficulty.

## 3. Discussion

A simple and technically easy method of correction of distal loop ileostomy prolapse is described in this case report. This minimally invasive procedure consumes the most commonly available instruments including linear stapler. The functional and structural outcome of the revised stoma is good. The intraoperative bleeding was minimal, and the surgery time was short. The patient had very mild postoperative pain, and the postoperative recovery was quick.

There are many surgical techniques described in the literature for uncomplicated stoma prolapse correction [7–11]. These techniques can be done by performing laparotomy and entry into the peritoneal cavity or local correction under local anesthesia. As this patient has presented with the gangrenous prolapsed segment, we preferred the surgery under general anesthesia with the anticipated conversion to laparotomy. The abdominal approach can result in morbidities. The local approach to revise the stoma prolapse intends to avoid these morbidities.

There are reported cases with minimally invasive stoma revision with a patent distal loop by using stapler devices [8]. In this patient, we preferred to do this distal loop closing method as the patient had just completed a month after the lower anterior resection and has to wait for another two months for ileostomy reversal.

## 4. Conclusion

Patients with stoma can develop complications like prolapse. Surgeons should be familiar with the minimally invasive revision for stoma prolepses. This stapler-assisted technique for stoma prolapse not only avoids unnecessary laparotomy and morbidity related to it but it also consumes less time and results in expedite recovery of the patient after surgery.
